# Prediction of Myoelectric Biomarkers in Post-Stroke Gait

**DOI:** 10.3390/s21165334

**Published:** 2021-08-07

**Authors:** Iqram Hussain, Se-Jin Park

**Affiliations:** 1 Center for Medical Convergence Metrology, Korea Research Institute of Standards and Science, Daejeon 34113, Korea; iqram@ust.ac.kr; 2 Department of KSB (Knowledge-Converged Super Brain) Convergence Research, Electronics and Telecommunication Research Institute, Daejeon 34129, Korea; 3 Department of Medical Physics, University of Science & Technology, Daejeon 34113, Korea; 4 AI Research Group, Sewon Intelligence, Ltd., Seoul 04512, Korea

**Keywords:** electromyography, physiological biomarker, gait, stroke, machine learning

## Abstract

Electromyography (EMG) is sensitive to neuromuscular changes resulting from ischemic stroke and is considered a potential predictive tool of post-stroke gait and rehabilitation management. This study aimed to evaluate the potential myoelectric biomarkers for the classification of stroke-impaired muscular activity of the stroke patient group and the muscular activity of the control healthy adult group. We also proposed an EMG-based gait monitoring system consisting of a portable EMG device, cloud-based data processing, data analytics, and a health advisor service. This system was investigated with 48 stroke patients (mean age 70.6 years, 65% male) admitted into the emergency unit of a hospital and 75 healthy elderly volunteers (mean age 76.3 years, 32% male). EMG was recorded during walking using the portable device at two muscle positions: the bicep femoris muscle and the lateral gastrocnemius muscle of both lower limbs. The statistical result showed that the mean power frequency (MNF), median power frequency (MDF), peak power frequency (PKF), and mean power (MNP) of the stroke group differed significantly from those of the healthy control group. In the machine learning analysis, the neural network model showed the highest classification performance (precision: 88%, specificity: 89%, accuracy: 80%) using the training dataset and highest classification performance (precision: 72%, specificity: 74%, accuracy: 65%) using the testing dataset. This study will be helpful to understand stroke-impaired gait changes and decide post-stroke rehabilitation.

## 1. Introduction

Stroke is one of the leading causes of disability and death in the elderly community [1]. Stroke happens due to brain-cell death in the absence of blood flow to brain cells. The blood flow is hampered due to rupture and the block in blood vessels. The neuro-electrical activity of the representative cortical lobe is disturbed due to this damage to brain cells and destabilizes the overall neural system. Acute ischemic stroke and intracerebral hemorrhage are the leading causes of neurological disorders among the elderly population, and affect millions of people with neurological deficits, physical disabilities, and dependent lifestyles [2,3]. An ischemic lesion affects the functional network architecture of cortical areas and hampers functional motor and cognitive outcomes [4,5,6]. Neurological impairment due to stroke contributes to disability, poor functional improvement, and lower quality of life. In addition, the cognitive deficit can reduce the usefulness of post-stroke rehabilitation and vastly increase the risk for psychological disorders such as depression and anxiety. Moreover, the economic burden of post-stroke treatment of patients with physiological impairment is significantly greater than those without.

Although significant advances have been made in early interventions to treat post-stroke patients, most survivors have remaining ambulation difficulties and hemiplegia. Hemiplegia generally reveals asymmetrical deficits in gait and is one of the most common disabilities observed in the post-stroke phase. Asymmetrical gait can result from muscle weakness, leading to incompetent mobility, lack of balance, and the threat of muscular wounds to healthy limbs [7,8]. Post-stroke recovery depends on neural adaptation and task-specific repetitive exercise according to the basics of neuroplasticity [9]. Frequent task-based ambulation can recover muscular injury and improve motor control and movement coordination in post-stroke patients.

Precise identification of aspects that predict cognitive and functional outcomes is needed for making clinical decisions, setting feasible targets and plans for rehabilitation, and directing patients accordingly. Functional motor and cognitive deficits are usual and persistent consequences of stroke and a significant factor responsible for physical dysfunction, slow physiological recovery, and a worse post-stroke lifestyle [10]. Conventional mental and neurological evaluations cannot be conducted immediately after stroke due to the medical aspects (e.g., fluctuating levels of arousal, pain, confusion, tiredness) and functional obstacles (e.g., sensory, linguistic, motor shortfalls) that hamper the patients’ capability to approach physiological examinations [11].

The physiological signal can be an effective tool of a real-time physiological monitoring system for early prognostics in daily life and a clinical environment [5,12,13,14,15,16]. Tracking muscular activity is essential for identifying gait impairment due to stroke [5,15]. Electroencephalography (EMG) is a non-invasive muscular activity monitoring method sensitive to irregularities of neuromuscular coordination caused by stroke. Several real-life applications of EMG have been investigated, such as an assistive tool for pedal-switching operation for elderly drivers [17] and controlling robotic hands [18]. During the rehabilitation of stroke patients, EMG changes can help to trace post-stroke patients in the non-clinical and clinical environments. Myoelectrical activity may vary with the selection of muscles of lower limbs. Understanding stroke-impaired changes in muscle and associated biomechanical properties guides post-stroke rehabilitation. In particular, the bicep femoris and the lateral gastrocnemius showed greater differences in myoelectrical activities of lower limb movement between stroke and control groups in previous studies [19,20].

EMG is the most widely used technique to gather information about the neuromuscular control of muscles and is commonly studied in research and clinical environments [21,22]. EMG is successfully practiced in clinics for diagnostic support [23], surgical interventions [24], and personalized rehabilitation protocols, including myoelectric control-based biofeedback [25], supporting clinical decisions [26], evaluating therapy, following up patients, evaluating fatigue [27], and supporting forensics medicine [28]. Researchers are successfully utilizing EMG to increase the quality of life of post-stroke patients. Surface EMG-based machine learning has been applied in gait-assistive robotic technologies [29,30] and treadmills [31] for motor rehabilitation, movement analysis for gait disorders, and recovery assessments [32]. Recently, EMG has been studied as an alternative brain–computer interaction (BCI) for detecting movement intention [33]. Moreover, feasibility studies have been performed to use EMG for muscle–computer interfaces [34] and human–computer interfaces [35]. EMG has also been investigated to assess post-stroke gait recovery. F. Infarinato et al. have reported EMG outcomes of eight post-stroke patients to understand the association of conventional therapy and robot-assisted gait training with changes in gait kinematics and EMG for functional gait recovery [29]. This study reported a significant difference in bilateral symmetry in the anterior tibialis muscles during the progression of rehabilitation and no significant changes in muscle activation patterns associated with functional gait recovery. Although most of the related studies reported non-significant alternations of muscle activity during the progress of gait rehabilitation [29,30,31,36,37], a few controversies have been reported for stroke patients [38] and spinal cord injury patients [39], showing significant changes in the myoelectric pattern.

Time-domain and frequency-domain analysis are most commonly used for surface EMG analysis to assess motor neurons and innervation zones and evaluate muscle contraction velocity [40]. Time-domain features, such as waveform length (WL), root mean square (RMS), and Willison amplitude (WAMP), have been utilized to develop the optimal feature vector [40,41]. Among frequency-domain analysis, mean frequency (MNF) and median frequency (MDF) are the most effective and widely used frequency-domain features for assessing muscle fatigue [40]. However, the frequency-domain power spectrum approach showed better accuracy to assess muscle fatigue [42]. Most of the studies reported either statistical and hypothesis tests or machine-learning analysis [43]. Simultaneous studies of statistical and machine-learning approaches have remained limited. Moreover, the majority of studies reported the outcomes of a small sample group. The machine learning model, developed with a small dataset, may cause overfitting problems, which could lead to flawed and misguided predictions. In addition, wearable sensors, big-data, and machine-learning-based cloud computing frameworks are becoming state-of-art technology in health monitoring [4], sleep monitoring [4,44], gait monitoring [5], driving monitoring [6], etc. Therefore, studies are needed to check the feasibility of an EMG-based gait monitoring system including wearable sensors, cloud data processing and a machine learning approach for muscular disorders, in particular, stroke-impaired gait.

We hypothesized that variations in the muscle activity due to the stroke-affected neuromuscular deficit would be acknowledged by the EMG measurements. Feature extraction using signal processing and dimensionality reduction, feature ranking, and machine learning (ML) methods would be a trustworthy technique for understanding the post-stroke muscular impairment of stroke patients.

We explored the neuro-electrical activity of the stroke group and the control group through EMG during gait. Our objective was to quantify EMG features for understanding myoelectrical changes because of stroke-derived physiological impairments and to identify the biomarkers to distinguish between stroke patients and healthy adults. The key contributions of this paper can be summarized as follows:

- We established an EMG-based neuromuscular disease prediction platform integrating the wireless EMG device, data streaming to a big data server, live signal processing in a big data platform, dashboards for the clients and clinicians for machine learning, and rule-based predictions of neuromuscular diseases;
- We investigated stroke-impaired EMG indices, including power spectrum features using statistical methods and hypothesis tests;
- We utilized the supervised machine learning algorithms to classify myoelectrical features of the stroke patients and the healthy adult group.

The rest of this study was organized into five sections. Section 2 described the experimental protocol and the methodology of the signal processing, statistical, and the machine learning analysis. The results are demonstrated in Section 3, followed by the discussion. Finally, the conclusions were narrated in Section 5.

## 2. Materials and Methods

### 2.1. EMG-Based Disease Prediction System

**Figure 1:**
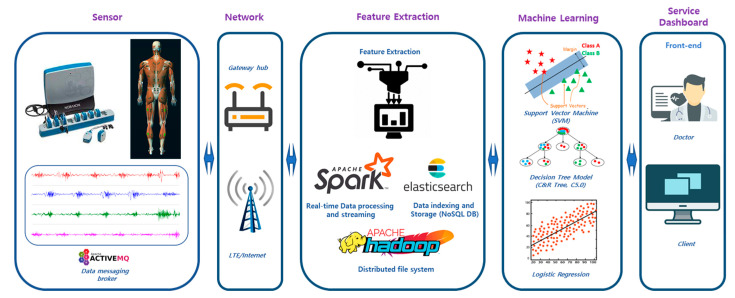
Overview of the EMG-based disease prediction system. The EMG data acquisition system consisted of a wearable EMG sensor. This system feeds the EMG data to a cloud server using Wi-Fi or LTE networks. In the cloud server, Elasticsearch indexes the data and acts as the No-SQL database. Spark performs feature extraction, feature selection, and machine-learning-based prediction and provides a query service for front-end service applications. This ambulatory system was developed to identify the changes in myoelectric features due to ischemic stroke or other disabilities and generate health advice to assist patients and hospitals.

An EMG-based neuromuscular health monitoring system was proposed to predict muscular diseases for the rehabilitation management of post-stroke patients. As demonstrated in Figure 1, this system consisted of wireless EMG sensors, the data transfer interface, the networking gateway, cloud storage and processing, and the physiological analytics service. The sensors generated electromyogram data as health level 7 (HL7 V2) messages, produced according to the protocol of the standards of HL7 International, to the Elasticsearch database (DB) through the Wi-Fi or LTE network [45]. Elasticsearch performs data indexing and stores the data in a No-SQL database. The feature extraction, feature selection, and machine learning algorithms were implemented in the Apache Spark platform for real-time processing. Wearable devices generate a vast amount of data and can be characterized in terms of 3Vs (volume, variety, and velocity) for big data [16]. These data need to be processed using big-data-based processing for real-time healthcare services. We utilized the Apache Hadoop platform for the online processing of wearable big health data [46]. Relevant muscular features, such as spectral power measures, were extracted using feature extraction algorithms. In the next step, feature selection found out the key features of diseases and reduced the computational time by eliminating unnecessary features for training the machine learning models. The selected neuromuscular features were fed to the ML model for training classification models. The medical knowledgebase showed the related muscular disorders or gait complications as assistance for rehabilitation and post-stroke treatment. All processed data could be visualized in dedicated monitors for patients, service providers, and assigned medical doctors.

### 2.2. Study Protocol

**Figure 2:**
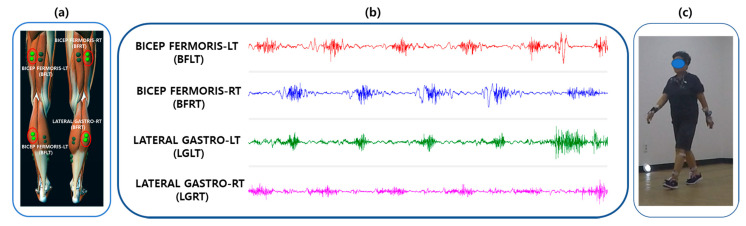
Electrode positions and layout of EMG data acquisition. (**a**) Four-channel EMG electrode positions based on bicep femoris muscles and lateral gastrocnemius muscles of both lower limbs. (**b**) EMG pre-processed signal recorded from four electrode positions. (**c**) An experimental scenario of a post-stroke patient.

This study was conducted according to a protocol approved by the Institutional Review Board of Korea Research Institute of Standards and Science, Daejeon, South Korea. Before the start of the experiment, the experimental scenario was explained to the participants. EMG electrodes were attached to the participants, connected to a wireless EMG device. At first, the participants were instructed to sit on a sofa for three minutes, followed by line-following walking. The subjects were instructed to walk along a designated line of a rectangular path inside the experiment hall. There was no definite walking duration, but subjects were allowed to finish the path of around 200 m walking in a natural way. As shown in Figure 2b, the study consisted of walking activities. EMG data were continuously recorded throughout the entire experiment. The temperature of the experiment room was kept at 24 °C and the relative humidity was maintained at 40%.

### 2.3. Demographics of Participants

Forty-eight stroke patients and seventy-five healthy adults were recruited for this study. The target group consisted of 48 stroke patients (mean age: 72.2 ± 5.6 years, 62% male). The control group comprised 75 healthy adults (mean age 77 years, 31% male). Both stroke and control groups were selected within the same age group range to decrease age-linked gait pattern variation. The participants comprised patients referred to the Stroke Rehabilitation Center, Chungnam National University Hospital, Daejeon, South Korea. Prior ischemic stroke occurrence of the patients was confirmed through MRI scans or CT (clinically). The control group comprised healthy adults without any history of ischemic or hemorrhagic stroke or known underlying gait disorders.

### 2.4. Data Acquisition

In this study, the EMG was acquired utilizing a Myoresearch DTS System (Noraxon Inc., Scottsdale, AZ, USA) and using a Noraxon MR3 Myomuscle software program. The surface EMG was recorded by applying disposable, wet-gel, self-adhesive Ag/AgCl snap dual electrodes to the left and right sides of the participants’ leg muscles connected to a wireless DTS EMG sensor with DTS EMG Pinch Lead wires (Common Mode Rejection Ratio > 100 dB, Gain: 500). The signals were transmitted to a 16-bit analog to digital (A/D) converter receiver (Noraxon DTS receiver) and saved to a computer at a sampling rate of 1500 Hz using MR3 software (Version 3.10.16, Noraxon USA Inc., Scottsdale, AZ, USA). Electromyography data were bandpass-Butterworth-filtered (high pass of 15 Hz and low pass of 450 Hz). A low-alcohol swab was used to clean the participants’ skin to reduce the impedance. As shown in Figure 2a, we only recorded EMG data taken on the two lower limb muscle positions: the bicep femoris muscle and the lateral gastrocnemius muscles of both left and right legs. Participants were recommended not to consume any drink such as coffee or alcohol and no physical exercise before the tests. During the collection of the EMG data, patients were instructed to walk in a normal manner. The raw four-channel EMG signals are displayed in Figure 2b. In addition, single-channel ECG was recorded for filtering the ECG artifact from the EMG signal. An experimental scenario of the EMG data acquisition is displayed in Figure 2c.

### 2.5. Pre-Processing

All recorded signals were manually inspected for artifacts, such as physiological signals, low-frequency motion artifacts, power line noises, or ADC clipping. The EMG signal was cleaned out of the 60 Hz AC noise of the local grid. The artifacts of electrocardiography (ECG) signals were filtered out of the EMG signal [47]. We used FastICA algorithms for denoising the EMG signal [48]. ICA utilizes ECG recordings to isolate EMG waveform from the cardiac artifacts. Low-frequency motion artifact noise was caused by movements of the wires attached to the EMG sensors and by the movement of the sensor relative to the skin. A signal-to-noise ratio (SNR) was estimated for each signal by taking the power ratio of the EMG signal and the second undisturbed measurement that was recorded immediately following the contraction [49,50]. If contamination was noticeable in the recorded signal or the SNR was below 18 dB, the signal was removed from the dataset [50].

### 2.6. Feature Extraction

The EMG frequency and power analysis extracted myoelectrical measures resulting from the power spectrum of an EMG signal. EMG waves were analyzed using the Welch periodogram estimation method, averaging time-divided portions of the signal and decreasing noise influence, and creating a 2D diagram displaying the power of a certain frequency component of a signal [51]. In this study, the fast Fourier-transform (FFT) method was applied to artifact-free EMG signals to determine the power spectral density (PSD) of the frequency components in the EMG signal. EMG signals were divided into fixed-width time epochs of 15 s each. Mean power, total power, median frequency, mean frequency, peak frequency of myoelectric waveform are considered as the standard measures for myoelectrical studies to find relationships between the disease-impaired and healthy muscular activity [52].

Median power frequency (MDF), measured in Hz, is the frequency where 50% of the entire power inside the epoch is reached. Mean power frequency (MNF), expressed in Hz, is the frequency where the average power inside the epoch is reached. Peak power frequency (PKF), measured in Hz, is the frequency where the peak power occurs within the epoch. Mean power (MNP), measured in V^2^/Hz, is the average power within the epoch. Total power (TP), expressed in V^2^/Hz, is the sum of the power at all spectral frequencies in the epoch. Mean power frequency and median power frequency were calculated according to Equations (1) and (2).

(1) $$\int_{0}^{\mathrm{MDF}}P\left(f\right)\mathrm{df}=\int_{\mathrm{MDF}}^{\infty }P\left(f\right)\mathrm{df}=0.5\int_{0}^{\infty }P\left(f\right)\mathrm{df}$$

(2) $$\mathrm{MNF}=\frac{\int_{0}^{\infty }\mathrm{fP}\left(f\right)\mathrm{df}}{\int_{0}^{\infty }P\left(f\right)\mathrm{df}}$$

where P is the power of EMG power spectrum, and f is the EMG frequency.

### 2.7. Feature Selection

Feature selection plays a vital role in high-dimensional biomedical data analysis. Classification performance largely depends on the relevance of features, and irrelevant or redundant data affect the computational power and time. Feature selection algorithms perform screening, ranking, and selection processes to find the most important features for a study. Screening removes feature variables, which do not provide useful information for prediction. Feature selection ranks the features based on the prediction accuracy of the individual variable. The Pearson chi-squared test measures the importance value of the predictor [53]. We evaluated the feature importance as (1 − p), where p is the chi-squared test outcome. We selected EMG features with a feature importance greater than 0.95 for training the machine learning algorithm.

### 2.8. Machine Learning Algorithms

Supervised machine learning techniques are an efficient tool for classification and discovering patterns in a dataset. In previous studies, machine learning has successfully been utilized. Machine learning and deep learning techniques are also utilized to classify multimodal physiological signals. Logistic regression, support vector machine (SVM), decision-tree-based machine learning algorithms, such as CART, C5.0, and biologically inspired neural network algorithms have been implemented to categorize myoelectrical features. We partitioned the EMG dataset into training and testing data. The training dataset comprised seventy percent of all feature data, and the test dataset comprised thirty percent of the entire feature dataset. We validated the trained models using k-fold cross-validation (k = 10).

#### 2.8.1. The Neural Network Model

Neural networks are data mining algorithms that make predictions based on growing multi-layered complex patterns [54]. We used the multilayer perceptron (MLP) neural network in this study. This model can estimate a wide range of analytical models with marginal demands on model structure and assumption. This model contains multiple input nodes, a neural network with hidden layers, and an output layer.

#### 2.8.2. C5.0 Model

The C5.0 model is a supervised data mining algorithms used to build decision trees or rule sets from data [55]. This model splits the data based on the field that provides the highest gain ratio. The model builds a decision tree, followed by a pruning procedure to minimize the tree’s estimation error rate. This model does not require a long training time for prediction and is robust to missing data and many input fields.

#### 2.8.3. Classification and Regression Tree Model

The Classification and Regression (C&R Tree or CART) Tree model is a classification and prediction method which splits the training data into a decision tree [56]. This method first grows the tree and then prunes it, based on a cost complexity algorithm. This model is stable in complications such as missing data and large numbers of input fields.

#### 2.8.4. Support Vector Machine Model

Support vector machine (SVM) is a widely used classification model that maps the data by forming a higher dimensional hyperplane so that features can be classified by creating a margin line using a popular kernel method, Gaussian Kernel or Radial Basis Function (RBF). We trained the SVM model and performed k-fold cross-validation (k = 10). SVM is most appropriate for use with wide-ranging datasets with lots of input fields [57]. The Gaussian Kernel function is defined in Equation (3).

(3) $$\mathrm{Gaussian}\;\mathrm{Kernel},\;K\;\left(X_{1}-X_{2}\right)=\mathrm{exponent}\left(-{\gamma ∥X}_{1}-X_{2}∥\right)$$

where ||X1 − X2 || is the Euclidean distance between two points, X_1_ and X_2_, and γ (gamma) is the inverse of the standard deviation of the RBF kernel (Gaussian function).

#### 2.8.5. Discriminant Analysis Model

The discriminant analysis model forms a predictive model for group relationships. The model comprises a discriminant function based on linear combinations of the predictor features that provide the best classification between the target groups.

#### 2.8.6. Logistic Regression Model

The logistic regression model is a statistical technique for classifying records based on values of input fields. This model works by forming equations that relate input variables to the probabilities associated with output classes. Once the model is trained, it can predict the classes of new data.

### 2.9. Statistical and Machine Learning Analysis

We analyzed and explored the myoelectrical data of the participants using descriptive statistics. The mean value with the error range of each feature was measured and presented in a bar chart. Data in the bar chart represent the mean value of each data point with a corresponding 95% confidence interval (CI). The independent-samples t-test was used as a comparative measure of the average of EMG features between the post-stroke patients and the healthy adults. A p-value of less than 0.05 was marked a statistically significant. Statistical analyses were performed using SPSS 26 software (IBM, Armonk, New York, NY, USA). We utilized machine learning algorithms to classify the post-stroke group and the control group. We partitioned the selected EMG feature datasets into two categories: the training dataset and the testing dataset. The training dataset was utilized in the machine learning algorithms to build the classification models, which were later utilized for prediction using the EMG testing datasets. We used IBM SPSS Modeler 18 software (IBM, Armonk, New York, NY, USA) for machine learning analyses.

## 3. Results

### 3.1. Statistical Investigation

We investigated the myoelectric features of the ischemic stroke group and control group using descriptive statistics to explore the changes in the electrical activity of the muscle. We also conducted the hypothesis test, such as the independent-samples *t*-test, to evaluate the statistical significance of variation of EMG features for two groups. This test performed a Levene’s test to measure the equality of variances and a t-test for the equality of means. The statistical significance was characterized as a *p*-value of less than 0.05.

#### Stroke-Impaired Myoelectric Biomarker

**Figure 3:**
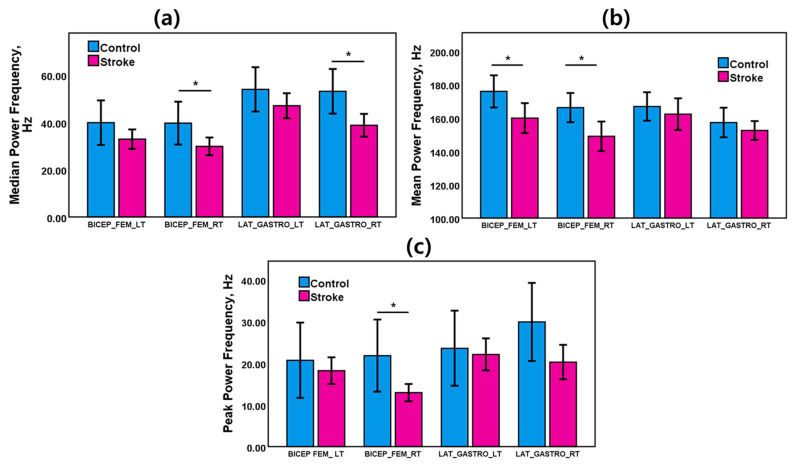
Bar charts with error bars of (**a**) median power frequency (MDF), (**b**) mean power frequency (MNF), and (**c**) peak power frequency (PKF) for the stroke group and the control group during the gait task. The bar charts represent the mean values, and the error bars show 95% confidence intervals (±95% C.I.) * (*p* < 0.05) indicates a significant difference (*t*-test).

EMG power spectra of the stroke and healthy control groups were evaluated during walking. Those myoelectric activities varied based on neuromuscular changes due to stroke. Figure 3a shows the mean and error bars with 95% confidence intervals of EMG frequency and power features of bicep femoris and lateral gastrocnemius muscles for left and right legs during the walking task. Table 1 presents the results of the statistical analysis of the EMG frequency and power features for the control group and the stroke group for gait tasks.

As shown in Figure 3a, EMG median power frequency was dominant in the control group. MDF of the stroke group was lower than the control group for BFLT, BFRT, LGLT and LGRT muscles. The MDF was 39.67 Hz for the control group and 29.83 Hz for the stroke group in BFRT muscle. The MDF was 53.12 Hz for the control group and 38.76 Hz for the stroke group in LGRT muscle. MDF of both right leg muscles (BFRT and LGRT) showed statistically significant differences between the stroke patients and the healthy adults.

As displayed in Figure 3b, a higher EMG mean power frequency was observed in the control group. MNF of the stroke group was lower than the control group for BFLT, BFRT, LGLT, and LGRT muscles. The MNF was 175.93 Hz for the control group and 160 Hz for the stroke group in BFLT muscle. The MNF was 166.21 Hz for the control group and 149 Hz for the stroke group in BFRT muscle. MNF of both bicep femoris muscles (BFLT, BFRT) showed statistically significant differences between the stroke patients and the healthy adults.

As shown in Figure 3c, EMG peak power frequency was also dominant in the control group. PKF of the stroke group was lower than the control group for BFLT, BFRT, LGLT, and LGRT muscles. The PKF was 21.80 Hz for the control group and 12.94 Hz for the stroke group in BFRT muscle. MNF of the right bicep femoris muscles (BFRT) showed a statistically significant difference between the stroke patients and the healthy adults.

Higher EMG mean power was seen in the stroke group. MNP of the stroke group was higher than the control group for BFLT, BFRT, LGLT and LGRT muscles (Table 1). The MNP was 0.0045 V^2^/Hz for the control group and 0.0273 V^2^/Hz for the stroke group in BFLT muscle. The MNP was 0.0072 V^2^/Hz for the control group and 0.0470 V^2^/Hz for the stroke group in BFRT muscle. The MNP was 0.0073 V^2^/Hz for the control group and 0.0250 V^2^/Hz for the stroke group in LGLT muscle. MNP of BFLT, BFRT and LGLT showed statistically significant differences between the stroke patients and the healthy adults.

EMG total power was also dominant in the stroke group. TP of the stroke group was higher than the control group for BFLT, BFRT, LGLT and LGRT muscles (Table 1). The TP was 4.66 V^2^/Hz for the control group and 27.96 V^2^/Hz for the stroke group in BFLT muscle. The TP was 7.35 V^2^/Hz for the control group and 48.13 V^2^/Hz for the stroke group in BFRT muscle. The TP was 7.48 V^2^/Hz for the control group and 25.67 V^2^/Hz for the stroke group in LGRT muscle. The total power of BFLT, BFRT and LGLT showed statistically significant differences between the stroke patients and the healthy adults.

### 3.2. Machine-Learning-Based Post-Stroke Gait Prediction

Receiver operating characteristic (ROC) analysis offers the most comprehensive description of predictions widely used in biomedical studies [58]. It shows all of the combinations of sensitivity and specificity that a machine-learning model can deliver. Area under the curve (AUC) is a performance indicator of the predictive model and defines the area under the ROC curve. The perfect score of the AUC is 1.0. A classification model with an AUC of less than 0.5 is not treated as an effective classifier. Another alternative measure of AUC is the Gini coefficient, ranging between zero and one, defined as two times AUC-1. The confusion matrix, or error matrix, delivers a complete representation of the predictions of true and false. We evaluated the standard classifier performance measures, including accuracy (ACC), sensitivity (true positive rate), specificity (true negative rate), precision (positive predictive rate), and negative predictive value from the confusion matrix. Accuracy was considered the most intuitive measure of performance to find the best model calculated as a percentage of the correct predictions across observations. Sensitivity is the true positive rate, defined as the correct positive predictive ratio of all actual observations. Specificity shows the true negative rate, characterized as the fraction of correct negative predictions to all actual observations. Model prediction outcomes can be presented using the following standard equations:

(4) Sensitivity=(True Postive)/(True Postive+False Negative)

(5) Specificity=(True Negative)/(True Negatve+False Positive)

(6) Precision=(True Positive)/(True Positive+False Positive)

(7) Negative predictive value (NPV)=(True Negative)/(True Negative+False Negative)

(8) $$\mathrm{Accuracy}\left(\mathrm{ACC}\right)=\frac{\left(\mathrm{True}\;\mathrm{Positive}+\;\mathrm{True}\;\mathrm{Negative}\right)}{\left(\mathrm{True}\;\mathrm{Positive}+\mathrm{False}\;\mathrm{Positive}+\;\mathrm{True}\;\mathrm{Negative}+\mathrm{False}\;\mathrm{Negative}\right)}$$

(9) $$\mathrm{Gini}\;\mathrm{Coefficient}=\sum_{i=1}^{m}p\left(i\right)\left(1-p\left(i\right)\right)\;$$

#### 3.2.1. Feature Selection Results

**Figure 4:**
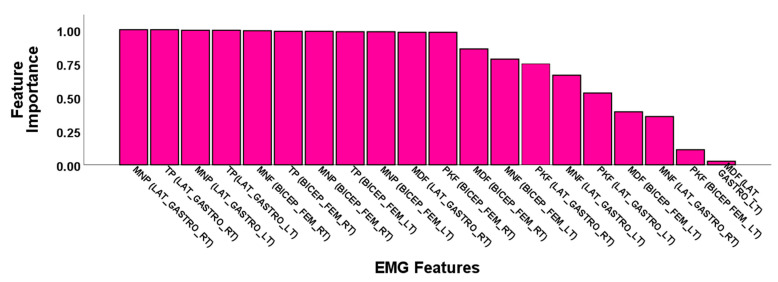
Feature importance of the EMG power spectrum features in the feature selection process of machine learning analysis to distinguish stroke and control groups.

The importance of electromyogram features resulting from the feature selection method are demonstrated in Figure 4. EMG fiducial features ranked higher than the feature importance of 0.95 chosen to train the ML models. EMG features were reduced to eleven features out of a total of twenty features for minimizing computational cost and improving the classification performance. Electromyogram power features were observed as dominant in feature importance.

#### 3.2.2. Classification Performance

**Figure 5:**
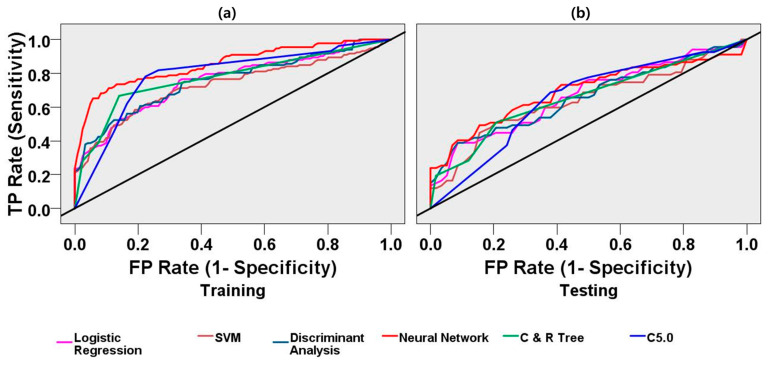
Receiver operating characteristic (ROC) curves for logistic regression, support vector machine (SVM), discriminant analysis, neural network, C&R Tree, C5.0 machine learning models for classification of the stroke group and the control group. (**a**) ROC curve using the EMG training datasets. The neural network model classified the training dataset with the highest AUC (85%) and accuracy (80%); (**b**) ROC curve using the EMG test datasets. The neural network model classified the test dataset with the highest AUC (69%) and highest accuracy (65%). The diagonal black line is the reference line.

The EMG feature data were partitioned into training and testing datasets. Training data belonged to 70% of the entire dataset, and testing data belonged to 30% of the entire dataset. Table 2 and Table 3 display all of the classifiers’ performance measurements for the training and test datasets. The neural network model showed the best accuracy in classification performance. The neural network model classified the training dataset with an AUC of 85%, accuracy (ACC) of 80%, and Gini-coefficient of 0.70. This model categorized the testing datasets with an AUC of 69%, accuracy (ACC) of 65%, and Gini-coefficient of 0.38. The neural network model showed the highest specificity (89%) and precision (88%) in the classification task using the training dataset. The C5.0 model showed the highest sensitivity (training: 78% and testing: 69%) and a negative predictive value (training: 76% and testing: 63%) in classification performance. This decision tree model classified the training dataset with an AUC of 79% and accuracy (ACC) of 78%. This model categorized the testing datasets with an AUC of 65% and accuracy (ACC) of 66%. The C&R tree model showed the highest specificity (79%) and precision (74%) in the classification task using the testing dataset. This decision tree model classified the training dataset with an AUC of 77% and accuracy (ACC) of 76%. This model categorized the testing datasets with an AUC of 66% and accuracy (ACC) of 64%. The logistic regression model classified the training dataset with an AUC of 75% and accuracy (ACC) of 71%. This model categorized the testing datasets with an AUC of 66% and accuracy (ACC) of 61%. The SVM model classified the training dataset with an AUC of 72% and accuracy (ACC) of 68%. This model categorized the testing datasets with an AUC of 64% and accuracy (ACC0 62%. The discriminant analysis model classified the training dataset with an AUC of 75% and accuracy (ACC) of 68%. This model categorized the testing datasets with an AUC 65% and accuracy (ACC) 59%. In Figure 5a,b, ROC curves demonstrate the classification models’ performance curves using the training and the test datasets.

## 4. Discussion

In our study, we aimed to characterize the electromyogram activity differences observed in post-stroke patients. The extent of muscular change due to stroke depends on the severity of the stroke and its consequent effect on neuromuscular activity. We have evaluated the myoelectrical features sensitive to post-stroke impairment during ambulation. The elderly adult volunteers possessed no history of physical muscular trauma or gait impairment; therefore, a healthy adult gait was characterized as the control condition. On the other hand, the post-stroke patients had experienced neuromuscular or gait abnormality to some extent, and post-stroke gait was explored to evaluate electromyogram biomarkers observed in the stroke group.

This variety in the activity of lower limb muscles during walking makes identification of the impairments of stroke challenging. Moreover, dissimilarities exist naturally between the left and right leg muscles. It is difficult to find a homogenous patient group in terms of stroke severity and period of the stroke, location of the brain lesion area, the process and period of post-stroke treatment, etc. Accurate lower limb muscle selection is essential for identifying stroke-impaired gait impairment. In this study, the bicep femoris and the lateral gastrocnemius muscles were investigated to understand the myoelectrical activities of lower limb movements between stroke and control groups. Although few studies have reported stiffness of the medial gastrocnemius in stroke patients, a controversial result was observed in another study [19]. MNF is usually higher than MDF for both post-stroke groups and healthy control groups for lower limbs [59]. This indicates that the power spectrum of the EMG signal was not extensively altered after a stroke. During the tests, participants were permitted to have adequate rest between experiments to reduce the effect of fatigue in the recording.

Significant changes in gait parameters were reported for the post-stroke patients. Researchers used statistical methods and machine learning approaches to evaluate the variations in gait and classify the gait features of post-stroke patients and healthy adults. C. Cui et al. demonstrated multi-modal physiological features (EMG, motion, and ground reaction force) and machine learning algorithms to discriminate between post-stroke and healthy gait [32,60]. Park et al. demonstrated the prediction of stroke-impaired gait using the ground reaction force and acceleration data through a machine learning approach [5,15]. J.C. Castiblanco et al. investigated the upper limb EMG measures of hand-impaired stroke patients, hand-non-impaired stroke patients, and healthy controls to classify myoelectric patterns using machine learning algorithms [61].

It has been identified in previous studies that a higher frequency of the power spectrum is an indicator of a superior muscle force [52,62]. It is observed that MNF was higher for the healthy adult group compared with the stroke group. Similar patterns were witnessed in the cases of MDF and PKF. Reduced MDF and PKF were observed for the stroke group compared with the healthy adult group. This finding is supported by the study of Angelova et al., who reported EMG power spectral statistical measures to understand the changes of myoelectric parameters due to stroke [62]. The mean power frequency (MNF), median power frequency (MDF), peak power frequency (PKF), and mean power (MNP) of the stroke group differed significantly from those of the healthy control group in our study. This outcome is in accordance with the study of Rasool et al., who investigated the spatial muscle activation patterns and reported a significant change in muscle architecture and morphology in stroke patients compared with healthy persons [63]. For the declining EMG activity patterns after stroke, the major contributors may include alterations in the overall morphology, micro-architecture, and functionality of skeletal muscles [64,65,66]. These changes can affect muscle functionality, inter-muscular synchronization, and overall motor functions. Although all stroke patients were taking extensive post-stroke rehabilitation treatment and no visible gait abnormalities were observed, a lack of neuromuscular coordination still existed due to stroke incidents. 

This study utilized logistic regression, discriminant analysis, SVM, neural network, C5.0, and C&R trees to classify the electromyogram patterns between stroke patients and healthy control volunteers. Machine learning and deep learning algorithms were utilized for early prognostics of diseases and classifications of patients with physiological disorders. Overall, the neural network model showed the highest accuracy, the highest AUC, and the highest Gini coefficient. As summarized in Table 4, other studies reflected that EMG power spectrum features effectively classified myoelectrical patterns between the stroke group and the healthy control group using statistical methods, machine learning, and deep-learning approaches. This finding is in accordance with the study by Cui et al. [32], who implemented machine learning techniques to assess post-stroke hemiparetic gait using multi-modal data including EMG. Researchers explored statistical and machine learning techniques for the accurate prediction of myoelectrical measures in post-stroke patients. Gaussian Naive Bayes classifier (GNB) and SVM were utilized for the classification of six hand motion patterns for controlling a robotic hand [18]. Moreover, linear discriminant analysis (LDA) was implemented for the classification of task-specific hand movements [67]. In another study, k-nearest neighbors (KNN), SVM, and LDA were used to identify the finger and hand motions for robotics-based rehabilitation [61]. Although prediction performances of few machine-learning models were not so impressive due to undergoing rehabilitation programs, most machine-learning models still predicted the stroke patients and healthy adults accurately.

In this study, we analyzed EMG measures based on only two muscular positions of lower limbs for understanding changes in EMG due to stroke impairment. The location of paretic muscle may vary among stroke patients; therefore, high-density EMG measurements may improve the prediction accuracy of paretic muscles for better stroke rehabilitation [68]. For this reason, this study generalizes to only the bicep femoris and the lateral gastrocnemius muscles with the current parameterization. Participants were instructed to walk steadily along a track. Several stroke symptoms started to be recovered due to the post-stroke treatment of patients during this experiment, which had an adverse influence on the classification accuracy of EMG features between the stroke group and the control group. Any kind of attempt of gait acceleration in walking may vary myoelectrical measures. In this study, we only explored the power spectrum features of EMG. In the future, we will investigate EMG-derived gait parameters, such as step length, stride length, and joint angles of lower limbs to evaluate the extended features to classify stroke groups and control groups.

## 5. Conclusions

An EMG-based neuromuscular disease prediction system, proposed here to predict stroke, can be used for the decision-making of post-stroke rehabilitation at home and the clinical environment. We explored the myoelectrical activity of stroke patients and healthy adults through EMG during motor tasks. The spectral power features were revealed as discriminative factors, classifying stroke patients and healthy adults in terms of the motor states of lower limbs. MDF and MNF were biomarkers for the stroke and the healthy control group during the motor tasks. The machine learning algorithms were also utilized to classify stroke patients and healthy adults using electromyogram features. This study will be helpful in the management of post-stroke treatment.

## Figures and Tables

**Table 1:** Results of the statistical analysis of the EMG features of the control and the stroke group. * Indicates *p* < 0.05.

EMG Features	Muscle	Control		Stroke		*t*-Test
		Mean	Standard Dev.	Mean	Standard Dev.	*p*-Value
Median Power Frequency (MDF), Hz	BICEP FEM. LT (BFLT)	39.87	63.75	32.90	29.36	0.17
	BICEP FEM. RT (BFRT)	39.67	61.43	29.83	26.78	0.04 *
	LAT. GASTRO. LT (LGLT)	53.94	63.66	47.03	37.78	0.19
	LAT. GASTRO. RT (LGRT)	53.12	64.01	38.76	34.50	0.01 *
Mean Power Frequency (MNF), Hz	BICEP FEM. LT (BFLT)	175.93	65.45	160.00	64.21	0.02 *
	BICEP FEM. RT (BFRT)	166.21	59.40	149.00	63.33	0.01 *
	LAT. GASTRO. LT (LGLT)	166.87	57.92	162.29	68.03	0.48
	LAT. GASTRO. RT (LGRT)	157.27	60.174	152.51	40.29	0.36
Peak Power Frequency (PKF), Hz	BICEP FEM. LT (BFLT)	20.69	61.06	18.20	22.98	0.59
	BICEP FEM. RT (BFRT)	21.80	58.40	12.94	14.92	0.04 *
	LAT. GASTRO. LT (LGLT)	23.57	60.89	22.09	27.29	0.76
	LAT. GASTRO. RT (LGRT)	29.86	63.38	20.25	29.44	0.06
Mean Power (MNP), V^2^/Hz	BICEP FEM. LT (BFLT)	0.0045	0.0040	0.0273	0.1178	0.01 *
	BICEP FEM. RT (BFRT)	0.0072	0.0116	0.0470	0.1921	0.01 *
	LAT. GASTRO. LT (LGLT)	0.0073	0.0197	0.0250	0.0794	0.01 *
	LAT. GASTRO. RT (LGRT)	0.0127	0.0237	0.0142	0.0696	0.78
Total Power (TP), V^2^/Hz	BICEP FEM. LT (BFLT)	4.66	4.15	27.96	120.72	0.01 *
	BICEP FEM. RT (BFRT)	7.35	11.94	48.13	196.93	0.01 *
	LAT. GASTRO. LT (LGLT)	7.48	20.21	25.67	81.46	0.01 *
	LAT. GASTRO. RT (LGRT)	12.98	24.32	14.52	71.36	0.78

**Table 2:** Results of the classification performance of different models using the training EMG dataset.

Model	Accuracy (ACC)	Sensitivity	Specificity	Precision	Negative Predictive Value	AUC	Gini
Neural Network	0.80	0.71	0.89	0.88	0.74	0.85	0.70
C5.0	0.78	0.78	0.78	0.79	0.76	0.79	0.57
C&R Tree	0.76	0.67	0.86	0.84	0.70	0.77	0.54
Logistic Regression	0.71	0.74	0.67	0.71	0.70	0.75	0.51
SVM	0.68	0.64	0.73	0.72	0.65	0.72	0.45
Discriminant Analysis	0.68	0.67	0.69	0.71	0.66	0.75	0.51

**Table 3:** Results of the classification performance of different models using the testing EMG dataset.

Model	Accuracy (ACC)	Sensitivity	Specificity	Precision	Negative Predictive Value	AUC	Gini
Neural Network	0.65	0.57	0.74	0.72	0.60	0.69	0.38
C5.0	0.66	0.69	0.62	0.68	0.63	0.65	0.30
C&R Tree	0.64	0.51	0.79	0.74	0.58	0.66	0.32
Logistic Regression	0.61	0.63	0.59	0.64	0.58	0.66	0.32
SVM	0.62	0.60	0.64	0.66	0.58	0.64	0.27
Discriminant Analysis	0.59	0.60	0.59	0.63	0.56	0.65	0.31

**Table 4:** Comparative study of methodologies and results between proposed study and previous studies.

Study	Study Sample	EMG Features	Findings	Application
Lu et al. [18]	Eight post-stroke subjects	Root mean square (RMS), 4th order auto regressive (AR) Coefficients, and waveform length (WL)	Mean classification accuracy, GNB): 84.8%; SVM: 83.3%; paired *t*-Test, *p*: 0.125	Classification of six hand motion patterns for controlling a robotic hand
Lee et al. [67]	Twenty stroke patients	Mean absolute value (MAV), the number of zero crossing (ZC), the slope sign change (SSC), and WL	Mean classification accuracy, LDA: = 71.3% for moderately impaired subjects.	Classification of task-specific hand movements
Castiblanco et al. [61]	Eighteen stroke patients and twenty-eight healthy control	MAV, RMS, SSC, MNF, mean power (MNP), MDF, and spectral moments (SM)	Accuracy classification of stroke and control group, KNN: 0.87; SVM: 0.82, and LDA: 0.74.	Identification of the fingers and hand motions for robotics-based rehabilitation.
Angelova et al. [62]	Ten stroke patients and fifteen healthy adults	Power spectrum features: MNF, MDF, Maximal power	MNF and MDF are lower for stroke patients compared with healthy control group.	Identification of changes in features during elbow flexion.
Proposed study	Forty-eight stroke patients and seventy-five healthy adults	MNF, MDF, PKF, TP, MP	Classification performance, neural network model: precision: 88%, specificity: 89%, accuracy: 80%.	Prediction of stroke-impaired myoelectrical changes through statistics and machine learning for understanding post-stroke impairment.
